# Acute myeloid leukemia presenting as unilateral central retinal vein occlusion

**DOI:** 10.3205/oc000202

**Published:** 2022-06-07

**Authors:** Ashok Kumar, Sandeep Shankar, Divya Kochhar, Amit Arora, Kapil Kumar

**Affiliations:** 1 Department of Ophthalmology, Armed Forces Medical College, Pune, India; 2 Department of Pathology, Armed Forces Medical College, Pune, India

## Abstract

Ocular manifestations of leukemia are often bilateral and involve all ocular structures with non-specific features like retinal hemorrhages, cotton wool spots and choroidal infiltrates. We report a rare, atypical initial presentation of acute myeloid leukemia with unilateral central retinal vein occlusion in a middle-aged male. This case will raise awareness among ophthalmologists to recognize and diagnose underlying systemic disease early and decrease systemic morbidity in consultation with a hematologist.

## Case description

**Figure 1:**
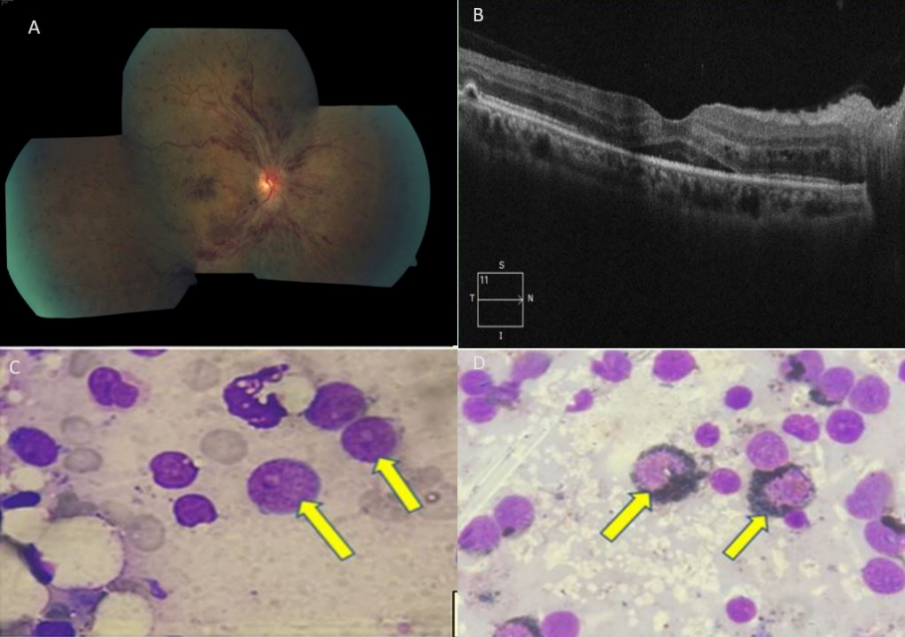
A) Fundus photo of the right eye showing dilated and tortuous retinal veins, optic disc edema and multiple flame-shaped, deep retinal hemorrhages involving posterior pole extending onto the retinal periphery suggestive of central retinal vein occlusion. B) Spectral domain optical coherence tomography (SD-OCT) of the same eye revealing subretinal fluid in the foveal region suggestive of cystoid macular edema. C) Bone marrow aspirate showing blasts, Leishman Giemsa stain, x1000 (yellow arrows). D) Blast cells showing myeloperoxidase (MPO) positivity (yellow arrows) with MPO stain, x1000 diagnostic of acute myelocytic leukemia.

A 45-year-old male with no associated systemic comorbidities presented to our ophthalmic outpatient department with profound diminution of vision in the right eye of five days duration. Best corrected visual acuity was 6/60 in the right eye and 6/6 in the left eye. The right-eye examination showed normal anterior segment with no evidence of inflammation in the vitreous cavity. Fundus examination revealed dilated and tortuous retinal veins, optic disc edema and multiple flame-shaped, deep retinal hemorrhages involving the posterior pole extending onto the retinal periphery suggestive of central retinal vein occlusion (Figure 1a (Fig. 1)). The macula showed presence of multiple hemorrhages with retinal thickening and edema. Fundus fluorescein angiography (FFA) showed a delay in arterio-venous transit time, and spectral domain optical coherence tomography (SD-OCT) revealed subretinal fluid in the foveal region suggestive of cystoid macular edema (Figure 1b (Fig. 1)). Examination of the left eye was completely unremarkable.

The complete blood count revealed pancytopenia with platelet counts of 18000/mm^3^, which made administration of intravitreal anti-vascular endothelial growth factor agent impossible. All other biochemical and coagulation parameters were normal. The patient was urgently referred to a hematologist to search for etiology of pancytopenia. Bone marrow aspirate showed trilineage hematopoiesis with an increase in lymphoid precursors and an M:E ratio of 5:1. Erythroid series showed normoblastic maturation, myeloid series showed maturation up to neutrophilic series. 30% medium-sized blasts were seen (yellow arrows) with a high N:C ratio, 1–2 nucleoli and basophilic cytoplasm with no Auer rods (Figure 1c (Fig. 1)). The blasts showed myeloperoxidase (MPO) positivity (yellow arrows) with MPO stain, thus confirming the diagnosis of acute myeloid leukemia (AML) (Figure 1d (Fig. 1)). The patient was immediately started on remission-induction chemotherapy by the hematologist and kept under close ophthalmic supervision in the form of comprehensive ocular evaluation as well as imaging in the form of SD-OCT. The best corrected visual acuity improved to 6/9 in the right eye with significant reduction in the macular edema as well as retinal hemorrhages without requirement of anti-vascular endothelial growth factors (anti-VEGF) or steroids.

## Discussion

Leukemias constitute a group of hematologic malignancies caused by acquired clonal abnormalities of hematopoietic stem cells that replace the normal bone marrow. Classically presenting with fatigue, fevers and bleeding, they are found to have ocular manifestations in 20 to 90% of affected patients, mostly being asymptomatic and bilateral [1], [2]. AML is the most common form of leukemia in adults, constituting around 40% of cases [3]. In the eye, leukemia can manifest in two forms: 1) as primary or direct infiltration of neoplastic cells (leukemic infiltrates and white centered retinal hemorrhages); and 2) as secondary or indirect involvement from nonviable or dysplastic cells, or from chemotherapy, leading to hematologic abnormalities (anemia, thrombocytopenia and hyper viscosity) and/or to immunosuppression causing opportunistic infections. The term ‘leukemic retinopathy’ describes the retinal manifestations of anemia, thrombocytopenia and hyper viscosity, rather than leukemic infiltration [1]. Common fundus findings in acute leukemia consist of preretinal and retinal hemorrhages because of anemia and thrombocytopenia, whereas hyper viscosity causing venous stasis is seen in chronic leukemia [3]. In our patient, the distribution of retinal hemorrhages in all quadrants, presence of optic disc edema and characteristic arterio-venous delay on FFA pointed towards vein occlusion rather than leukemic retinopathy alone.

Pulmonary and cerebral leukocytosis are more well-known early complications of AML than ocular involvement [4]. Bilateral simultaneous central retinal vein occlusion was reported by Tseng et al. in a young male, but our case had unilateral involvement with no manifestation in the other eye [5]. Bilateral CRVO in young males more often points to a systemic abnormality in the form of hyperhomocysteinemia, multiple myeloma, coagulation disorders or leukemia rather than in unilateral presentation. In the presence of pancytopenia at onset, we hypothesize that leukostasis or leukemic infiltration of the retinal vasculature could have resulted in unilateral central retinal vein occlusion. The patient was immediately started on remission-induction chemotherapy under the observation of the hematologist and under ophthalmic supervision. The patient did not show any features of involvement of the other eye on follow-up.

## Conclusion

Hematological malignancies can have varied ocular presentations, and often ophthalmologists are the first clinicians to examine and diagnose them. A thorough ophthalmic and systemic examination, especially in unusual presentation like ours, can help in the early diagnosis and effective treatment of this life-threatening illness in conjunction with the hematologist.

## Notes

### Competing interests

The authors declare that they have no competing interests.

## References

[1] Talcott KE, Garg RJ, Garg SJ (2016). Ophthalmic manifestations of leukemia. Curr Opin Ophthalmol.

[2] Reddy SC, Jackson N, Menon BS (2003). Ocular involvement in leukemia – a study of 288 cases. Ophthalmologica.

[3] Schachat AP, Markowitz JA, Guyer DR, Burke PJ, Karp JE, Graham ML (1989). Ophthalmic manifestations of leukemia. Arch Ophthalmol.

[4] Lee YH, Lee JY, Kim YS, Kim DH, Kim J (2006). Successful anticoagulation for bilateral central retinal vein occlusions accompanied by cerebral venous thrombosis. Arch Neurol.

[5] Tseng MY, Chen YC, Lin YY, Chu SJ, Tsai SH (2010). Simultaneous bilateral central retinal vein occlusion as the initial presentation of acute myeloid leukemia. Am J Med Sci.

